# Two Dimensions of Moral Cognition as Correlates of Different Forms of Participation in Bullying

**DOI:** 10.3389/fpsyg.2021.768503

**Published:** 2022-02-18

**Authors:** Simona C. S. Caravita, Johannes N. Finne, Hildegunn Fandrem

**Affiliations:** ^1^Norwegian Centre for Learning Environment and Behavioural Research in Education, University of Stavanger, Stavanger, Norway; ^2^Department of Psychology, Catholic University of the Sacred Heart, Milan, Italy

**Keywords:** moral disengagement, moral domains, bullying, defending the victim, passive bystanding

## Abstract

The present study investigated the extent to which moral disengagement and the tendency to consider moral rules as socio-conventional rules are distinct dimensions of morality, and their association with three different forms of participation in bullying (perpetrating bullying, defending the victim and passive bystander behavior). These two types of moral cognitions have been theorized in different models of morality and are usually studied independently, even if research on *moral shifts* (the interpretation of a moral rule transgression as a socio-conventional rule transgression) suggests some possible overlaps. A group of 276 Italian students from primary and middle school (aged 8–15) completed self-reports assessing moral disengagement, socio-conventional perception of moral rules, and participation in bullying as bully, defender of the victim and passive bystander. Results from structural equation modeling analysis confirmed that moral disengagement and socio-conventional comprehension of aggressions are separate and moderately connected morality dimensions. Controlling for age, gender and SES, only moral disengagement was positively associated with perpetrating bullying. These results point to moral disengagement as the critical component of moral cognitions to be addressed in interventions.

## Introduction

Research has devoted considerable attention to processes explaining the associations between moral cognitions and bullying behavior, and two theoretical perspectives on morality have been mainly used: (i) using mechanisms of self-justification that allow the person to act in an aggressive way without feeling guilty by cognitively restructuring the situation, namely *moral disengagement* (Bandura, 1991); (ii) judging moral rules forbidding to harm others as breakable because of a wrong conception of them as dependent on the authorities’ statements, and, by consequence as non-worthy by themselves, so that their transgression can be accepted (*social domain theory*; Turiel, 1983).

There is evidence that also bullying perpetrators can evaluate bullying as wrong (Gasser and Keller, 2009); nevertheless they bully peers. Both the social domain and the moral disengagement theories provide some explanation of this gap between the moral evaluation and the actual perpetration of bullying. Extensive literature (e.g., Killer et al., 2019) has provided evidence that higher moral disengagement is associated with higher bullying perpetration and lower defending, and in some studies with higher passive bystanding (Gini, 2006). In the few studies on the perception of bullying as a socio-conventional rule transgression, understanding bullying as violation of socio-conventional rules has been found to be associated with increased bullying perpetration and lower defending (Caravita et al., 2012).

The theoretical frameworks supporting these two types of moral knowledge are different, and they may be conceived as distinct moral mechanisms. Nevertheless, scarce literature has examined these two constructs in the same framework (Caravita et al., 2012; Thornberg and Jungert, 2013). The phenomenon of the *moral domain shift* (Leenders and Brugman, 2005) suggests the possibility of their inter-connection. If they may relate to each other, however, they may relate differently to forms of participation in bullying when they are considered in the same framework. In this study we aim to contribute to fill in this gap in the literature, by investigating to what extent these two types of moral knowledge are related to each other and to forms of participation in bullying, in order to further light on the organization of moral mechanisms in relation to social behaviors.

### Moral Disengagement

Moral disengagement (Bandura, 1991) refers to social cognitive processes through which the person can commit actions that they evaluate to be wrong, by cognitively restructuring the events and selectively avoiding moral censure.

Moral disengagement activates four clusters of cognitive processes aimed at: (1) redefining one’s own behavior according to personal purposes; (2) displacing personal responsibility for one’s own conduct to other persons or within the group; (3) minimizing the behavior consequences; and (4) considering the victim responsible for the situation or denying the victim’s human characteristics. The motivation of activating moral disengagement comes from the need to solve the cognitive dissonance (Festinger, 1957), as the uncomfortable inner state stemming from inconsistencies between one’s own actions, beliefs, attitudes or feelings. Moral disengagement mechanisms are used to reduce this uncomfortable inner state due to evaluating one’s own behavior as wrong. Moral disengagement has also been conceptualized as learnt socially. It first acts as an *a posteriori* mechanism, after the perpetration of the transgression, as the child learns from other social agents to (self-) justify what they did in order to avoid the subsequent guilt or shame feelings (Bandura, 1986). Then, with the use, moral disengagement starts to be used in the *while* or *before* perpetrating the transgressive action. Accordingly, early adolescents can learn to use moral disengagement from their peers (Caravita et al., 2014). With reference to school bullying, higher levels of moral disengagement are associated with increased bullying (Killer et al., 2019), and lower defending of the victims (Jiang et al., 2020). In some studies, moral disengagement was associated also with higher passive bystanding (Jiang et al., 2020), but the research on this behavior in bullying is still scarce and inconsistent (Mazzone et al., 2016).

### Social Domains

From a different theoretical perspective, Turiel’s (1983) social domain theory proposes that the social cognition is organized in separate domains. Persons’ social experiences influence the development and organization of their social knowledge in varying domains referred to the rules that allow or forbid social behaviors. The basic domains have been defined as *moral* (about concepts on fairness, rights, harm and welfare), *socio-conventional* (about concepts on social organization, social systems, and social conventions), and *personal* (about concepts on persons, self, identity and internal states) (Smetana, 1995). An important finding is that people consider transgressions in the moral domain as more serious and less acceptable than transgressions in the socio-conventional and personal domain (Nucci, 2001).

The moral domain is constructed and developed through experiences of actions that have negative or positive effects on the welfare of others or oneself. The socio-conventional domain, instead, refers to actions regulated by rules thought to depend on authorities’ statement (context authority’s dependence) and not on superior moral values, they are considered non-universally valid and their transgressions are evaluated less serious than breaking moral rules. The organization of moral knowledge in latent domain structures, informing and influencing the social information processing, has been hypothesized to be shaped by the repeated social interactions (Arsenio and Lemerise, 2004). After established, these mental structures may act *a priori*, leading the action.

### Moral Disengagement and Social Domains in Relation to Bullying

Compared to peers, bullying perpetrators also evaluate moral rule transgressions as wrong (Gasser and Keller, 2009). Nevertheless, in early adolescence they are more prone to consider both moral and socio-conventional rules as dependent of the context, thus to attribute also moral rule transgressions to the domain of socio-conventional rules (Caravita et al., 2009). Furthermore, considering breaking moral rules as acceptable (thus as socio-conventional rule transgression) has been found to be associated with higher bullying (Caravita et al., 2012). To our knowledge, no studies have examined the *understanding* of bullying as breaking of a socio-conventional rule in relation to passive bystanding, even if this behavior was associated with higher recognition of bullying as harming the victim and empathizing with the victim (Thornberg and Jungert, 2013).

In general, very few studies have investigated moral disengagement and social domains in the same framework in relation to youth’s behavior in bullying (Caravita et al., 2012; Thornberg and Jungert, 2013). Both these two moral dimensions may be relevant in explaining behaviors in bullying, and they are possibly related to each other, even if they were conceptualized within separate theoretical frameworks. In a community sample of adolescents Leenders and Brugman (2005) found that a *domain shift* from the moral toward non-moral (socio-conventional and personal) domains appeared when the adolescents evaluated hypothetical situations about delinquent behavior. That is, they attributed the delinquent behaviors that they perpetrated, but not other delinquent behaviors, to non-moral rule domains. This domain shift, only emerging in relation to one’s own transgressions, may serve a similar function as moral disengagement, by legitimizing one’s own transgressive behaviors, reducing the cognitive dissonance and preserving the self-esteem. This theorization about domain shifts suggests a possible relation, if not partial overlap, between the organization of moral knowledge in domains and the moral disengagement mechanisms. Nevertheless, the scarcity of research on the association between the two constructs does not allow to establish to what degree they are inter-related and their relative weights in explaining social behaviors.

### The Current Study

In this study we investigated the intertwin of moral disengagement and the perception of aggressions as socio-conventional rule transgressions in association with perpetrating bullying, defending the victim and passive bystander behavior in bullying, focusing on late-childhood and early adolescence as critical age levels (Caravita et al., 2009, 2012).

Our first purpose was to better investigate whether and to what extent moral disengagement and the understanding of aggressions as socio-conventional rule transgressions are related expressions of the moral knowledge organization. We hypothesized that these two morality components are mainly distinct, as the social domains should work more as a static *a priori* (before the action) organization of the socio-moral knowledge, while moral disengagement should be a more dynamic process, at the beginning working *a posteriori*, after the perpetration of the action. Nevertheless, as both these constructs should emerge from social interactions (Bandura, 1986; Arsenio and Lemerise, 2004), and may show some possible overlap in their functioning, (moral shifts; Leenders & Brugman), we hypothesize that they can be inter-correlated. We examined these hypotheses by running a structural equation model (SEM) in which the two moral dimensions were indicators of two separate latent factors, tested against a model in which they were indicators of only one latent factor of morality.

Our second purpose was to investigate whether these two components of the morality differently predict three main forms of participation in bullying, as bully, defender of the victim, and passive bystander. Based on the literature, we hypothesized that both these moral dimensions are associated with higher bullying perpetration and lower defending, while the literature on passive bystanding is too scarce to formulate clear hypotheses. As only few studies have considered the two dimensions of morality in the same framework, we cannot formulate clear hypotheses as well on which of the two dimensions is the most relevant in explaining participations in bullying. Nevertheless, the consistency of the literature on moral disengagement and bullying suggests that this mechanism may be the most important.

## Method

### Participants

Participants were 276 fourth to eight-graders (8–15 years; *Mage* = 11.21; *SD* = 1.52; 50% girls), attending one primary school (44.2%, *Mage* = 9.80, SD = 0.68) and one middle school (55.8%, *Mage* = 12.31, SD = 1.01) in Northern Italy. Participants’ majority (85.9%) had an Italian background. To assess the socioeconomic/cultural status (SES) participants reported their parents’ jobs and qualifications: 32.9% had families of low-average SES. 32.2% of average SES and 31.8% of average-high SES; 3.1% were not able to provide this information.

### Measures

#### Moral Disengagement Scale

We administered a self-report questionnaire specifically devised to assess moral disengagement in bullying situations (Caravita et al., 2011; Oliveira et al., 2019). The measure was an adaptation of the scale for children by Caprara et al. (1995). It consisted of 17 items assessing seven of the eight mechanisms of moral disengagement (the mechanisms assessed in the version for children of the original adapted measure): moral justification, euphemistic language, advantageous comparison, displacement of responsibility, distorting consequences, dehumanization of the victim, attribution of blame to the victims. Each measure item presented a statement of moral exoneration of bullying conduct (e.g., “Hitting annoying classmates is just like giving them a lesson”; 5 point response scale: 1 strongly disagree to 5 strongly agree). Higher scores indicated higher moral disengagement for bullying. We performed a second-order Confirmatory Factor Analysis (estimator MLR; Mplus 8.4, Muthén and Muthén, 1998–2017) to test the structure of the scale according to Bandura’s (1986) theoretical model, with parcel scores of the seven mechanisms loading the four moral disengagement clusters, which in turn loaded a unique latent factor of moral disengagement. The model fitted the data well: χ^2^(12) = 8.422 *p* = 0.751, *CFI* = 1.000, *RMSEA* = 0.000 (90% CI.000.044), moral disengagement factor Chronbach’s alpha = 0.78.

#### Socio-Conventional Perception of Moral Rules on Aggressions

A measure adapted by the scale developed by Caravita et al. (2012) was administered. The measure included 16 items, consisting in scenarios where a school socio-conventional rule (4 stories) or a moral rule (12 stories) was broken. The 12 scenarios describing the break of a moral rule (preserving other’s wellbeing) regarded situations of aggressive behaviors of three types: physical (4 scenarios), verbal (4 scenarios) and relational aggressions (4 scenarios). For the present study only the 12 scenarios on aggressions were considered. In all the items the main character was a student breaking a school rule. In half of the scenarios characters were girls and in half boys. For each scenario the respondent evaluated on a 4 point likert scale (1 = totally wrong to 4 = totally right) whether the character’s behavior is acceptable under three socio-conventionality conditions: if allowed by the principal (main school authority dependency), if allowed by the class teacher (class authority dependency), if behaved out of school (context dependency). Higher scores indicated higher perceived socio-conventionality. We confirmed the structure of the scale by means of a second-order CFA, with parcel scores of the three conditions as manifest indicators of the latent factors of the three types of aggressions, which were indicators of the overall socio-conventionality attributed to the moral rules: model fit χ^2^(15) = 22.956 *p* = 0.085, *CFI* = 0.995, *RMSEA* = 0.044 (90% CI.000.078), socio-conventionality factor Chronbach’s alpha 0.96.

#### Forms of Participation in Bullying

We assessed forms of participation in bullying as perpetrator, defender, and passive bystander by administering a peer report measure adapted from Pozzoli et al.’s (2012) scale. Each of the three behaviors was assessed by three items describing the participation form in situations of verbal, physical and relational bullying. The respondent had to nominate the classmates who more often behaved the way described (unlimited peer nominations). A definition of bullying was provided at the beginning of the questionnaire. Per each item the sum of the received peer nominations was standardized among classmates. The participation behavior score was the item average. Chronbach’s alphas: bully 0.78, defender 0.84, passive bystander 0.68.

### Procedure

School principals and teachers’ committees authorized the participation of some classrooms in grades four and five (last two grades of primary school), and six to eight (middle school). Participants’ parents/legal guardians authorized students’ participation in the study by providing active consent in response to a letter describing the study and its aims. The 72.3% of families authorized their children participation. Measures were group-administered in classroom situations, during the regular school hours, by a researcher assistant who discussed with the participants the definition of bullying and answered their questions. Participants were informed that they could withdraw from the study at any time without providing any explanation.

### Strategy of Analysis

**FIGURE 1:**
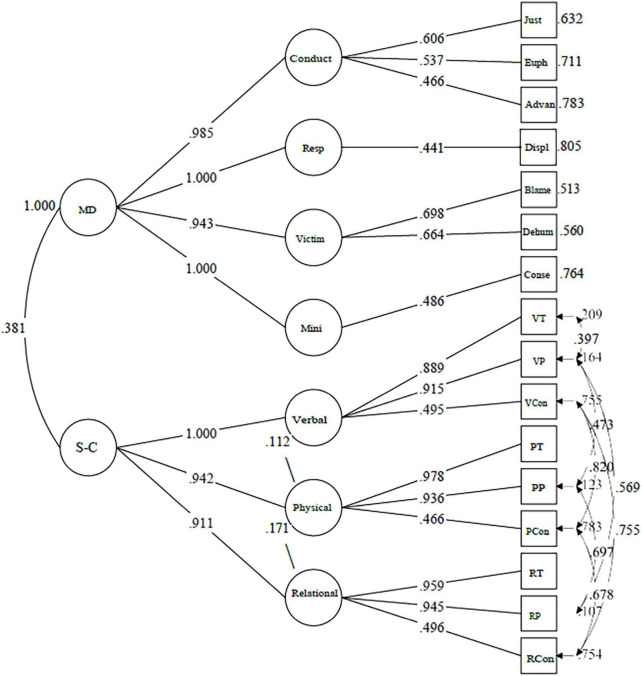
Moral dimensions, two latent factors model. Only significant paths (*p* < 0.05) are displayed in the figure. Standardized indices. MD, Moral Disengagement, S-C, Socio-conventionality of the moral rules for aggression; Conduct, conduct restrictuding mechanisms, Resp, responsibility restructuring mechanisms, Victim, victim restructuring mechanism, Mini, consequence minimization moral disengagement mechanism, Just, moral justification mechanism, Euph, euphemistic labeling mechanism, Advan, advantagious comparison mechanism, Displ, responsibility displacement mechanism, Blame, victim blame mechanism, Dehum, dehumanization mechanism; VT, verbal violence teacher’s permission, VP, verbal violence principal’s permission, VCon, verbal violence context dependence; PT, physical violence teacher’s permission, PP, physical violence principal’s permission, PCon, physical violence context dependence; RT, relational violence teacher’s permission, RP, relational violence principal’s permission, RCon, relational violence context dependence.

We used Structural Equation Modeling (Robust Maximum Likelihood MLR estimator, MPlus 8.0, Muthén and Muthén, 1998–2017) to test our hypotheses. As first step, we run a set of Exploratory Factor Analyses (EFAs; one to seven factors, factor axes extraction method, Geomin obliquation rotation) to examine the dimensionality emerging from the four clusters of moral disengagement and the nine parcel scores of the aggression conventionality. Then, we run Confirmatory Factor Analyses (CFAs) to test two competitive models: the one-factor model of morality, in which the latent factors of the four clusters of moral disengagement and the three latent factors of the socio-conventional perception of physical, verbal and relational aggressions were specified as indicators of one second-order latent factor of morality; the two factor model in which the two latent factors of moral disengagement and the socio-conventional perception of aggression were separate morality latent factors (Figure 1).

As third step, we tested a SEM model in which the three forms of behaviors in bullying were regressed on the final latent structure of morality emerging from step two. Gender (*one* boy, *two* girl), SES and age were included among predictors to control for their effects.

The model goodness of fit was examined against these indices: Chi-square (χ^2^), which needs to be non-significant for good fitting models, but which is also sensitive to the sample size and tends to become significant for large samples; the *CFI* index, with a value ≥0.90 for acceptable fit (Bollen, 1989) and ≥0.95 for good fit (Hu and Bentler, 1995); the *RMSEA* index with a value ≤0.08 for acceptable fit and ≤0.05 for good fit. Comparison between competitive models was also based on the Chi-square difference test for nested models, retaining the model with the best fit and if the decrease of the Chi-square was significant. The appropriateness of the sample size was established by running a power analysis, which showed that for testing a model with 180 degrees of freedom (our most complex model) and expecting a value of RMSEA equal to 0.08, a sample of 200 participants was enough to provide a power of 1.00 (MacCallum et al., 1996). Furthermore, the criteria recommended for performing CFAs (with *p* variables, *N/p* should be ≥10; Marsh et al., 1998), indicated in 230 the minimum participants’ number to test a CFA model including 23 variables (our two-factor second-order CFA).

## Results

### Moral Knowledge Organization

The EFAs indicated a six factor model as the best model of parcel scores (Supplementary Material): χ^2^(39) = 33.937 *p* = 0.670, *CFI* = 1.000, TLI = 1.000, *RMSEA* = 0.000 (90% CI.000.033), 5 factor model χ^2^ Difference test (11) = 88.975 *p* = 0.000. One factor (the fourth extracted factor), was loaded only by the seven parcel scores of moral disengagement. The remaining five factors expressed the dimensionality of the measure assessing the socio-conventionality of aggressions. The first extracted factor expressed the authority dependence and was positively loaded by the six indicators of teacher and principal dependency. The three scores of context dependency characterized the second extracted factor. The third extracted factor was loaded positively by teacher dependency of verbal aggression and negatively by the principal dependency of the physical aggression, thus mainly expressing the different perception of seriousness of these two forms of aggression. The fifth extracted factor was mainly loaded by the teacher dependency (0.661) and the principal dependency (0.295) of relational aggression, thus expressing the specificity of this form of aggression. The sixth extracted factor was mainly characterized by principal dependency of verbal (0.247) and relational violence (0.221), expressing some residual variance explained by this type of authority. The moral disengagement factor correlated moderately and significantly with first (0.301, *p* < 0.05), second (0.463, *p* > 0.05), and fifth extracted factors (0.220, *p* < 0.05) and non-significantly with third factor (0.014). On summary, EFAs provided a first indication that moral disengagement and conventionality of aggression are distinct morality dimensions.

In the CFAs, the one-factor model of morality had a non-adequate fit: χ^2^(90) = 263.730 *p* = 0.000, *CFI* = 0.934, *RMSEA* = 0.084 (90% CI.072.095). The two-factor model fitted the data adequately, with significantly lower Chi-square than the one-factor model: χ^2^(89) = 136.920 *p* = 0.000, *CFI* = 0.982, *RMSEA* = 0.044 (90% CI.029.058), χ^2^ Difference test with Satorra–Benter correction (1) = 94.307 *p* = 0.000. Then the two-factor model was retained as the final morality model (Figure 1). The two dimensions of morality were moderately associated (0.381).

### Moral Dimensions and Participant Roles Behavior in Bullying

**FIGURE 2:**
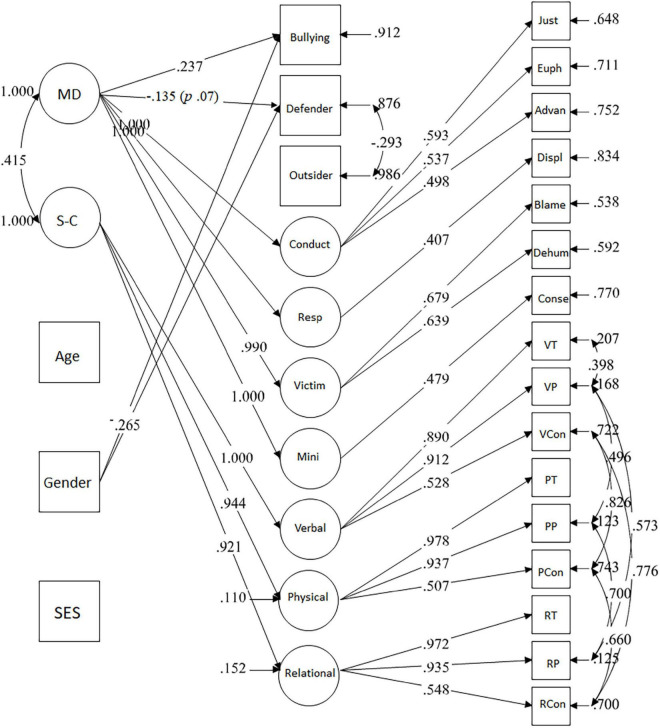
Moral dimensions and forms of participation in bullying. Only significant paths (*p* < 0.05) are displayed in the figure. Standardized indices. Md, Moral Disengagement, S-C, Socio-conventionality of the moral rules for aggression; Conduct, conduct restrictuding mechanisms, Resp, responsibility restructuring mechanisms, victim, victim restructuring mechanism, Mini, consequence minimization moral disengagement mechanism, mJust, moral justification mechanism, Euph, euphemistic labeling mechanism, Advan, advantagious comparison mechanism, Displ, responsibility displacement mechanism, Blame, victim blame mechanism, Dehum, dehumanization mechanism; VT, verbal violence teacher’s permission, VP, verbal violence principal’s permission, VCon, verbal violence context dependence; PT, physical violence teacher’s permission, PV, physical violence principal’s permission, PCon, physical violence context dependence; RT, relational violence teacher’s permission, RP, relational violence principal’s permission, RCon, relational violence context dependence.

The model (Figure 2) in which the two morality dimensions predicted the variance of behaviors in bullying had an adequate fit: χ^2^(180) = 282.201 *p* = 0.000, *CFI* = 0.965, *RMSEA* = 0.048 (90% CI.037.058). Bullying was higher for boys and defending for girls. Older youths showed higher defending. Moral disengagement was positively associated with bullying perpetration and marginally (*p* = 0.067) negatively with defending. The socio-conventional perception of aggressions was not associated with any of the three behaviors.

## Discussion

Goal of this study was to investigate whether and to what extent moral disengagement and the socio-conventional perception of aggressions (moral rule transgressions) are possibly related dimensions of morality, and their associations with participation in bullying as bullying perpetrator, defender of the victim, and passive bystander. Our findings confirmed that moral disengagement mechanisms and the socio-conventional perception of aggressions are distinct dimensions of morality, only moderately associated. Only moral disengagement was associated with higher perpetration of bullying and (marginally) with lower defending.

The distinction between the two moral dimensions may relate to the fact that social domains may be a more static organization of moral knowledge, mainly working *a priori*, before the perpetration of the action, while moral disengagement is a mechanism working (originally) *a posteriori*, after the transgression perpetration, to avoid negative emotions. This difference between the two dimensions can be particularly evident in the case of bullying. Students who bully others are aware of the importance of other’s well-being and they know that bullying is wrong (Gasser and Keller, 2009); yet they perpetrate bullying and use moral reasons less frequently and are less prone to referring to the victim’s harm in their judgments on bullying (Thornberg et al., 2017). Our findings suggest that the moral distortions of bullying perpetrators may lay more in the *a posteriori* self-justification of behaviors that they know to be wrong than in the *a priori* distortion in the organization of the knowledge about social behaviors in domains. The main difficulty about bullying perpetration, therefore, may lay in motives other than a static organization of moral knowledge, in which bullying is interpreted as a behavior that can be allowed under some circumstances (as a socio-conventional transgression), in that the distinction may be blurred in people’s mind, or can vary significantly depending on the person (Kelly et al., 2007; Margoni and Surian, 2021). Bullying has been consistently found to be related to a high popularity peer status, bullies’ motivation to achieve and keep dominance among peers (Caravita and Cillessen, 2012; Thornberg and Delby, 2019), peer norms (Salmivalli and Voeten, 2004) and perceived peer pressure as well (Juvonen and Galván, 2008). These factors may be the relevant motives that lead youth to behave bullying, but, as they evaluate bullying wrong, they need to use moral disengagement to avoid the subsequent negative feelings.

Moral disengagement, however, was significantly related only with bullying perpetration. Consistent with previous studies (Gini et al., 2014: Thornberg et al., 2021), a negative, but marginal, association appeared between moral disengagement and defending. This outcome may indicate that moral factors other than moral reasoning may be more relevant to explain defending. Eisenberg et al. (2016) suggested that there are several motives for performing prosocial behaviors, including egoistic concerns (e.g., the expectation of reciprocity), practical concerns (e.g., preventing an unwanted situation or helping), and other-oriented concerns (e.g., sympathy). Defenders also show high levels of moral sensitivity to the distress of victims, which may lead to higher sympathetic emotions (Menesini et al., 2003). Therefore, for defending the victim, the most important dimensions of the moral functioning may be more related to emotions than cognitive mechanisms.

Passive bystanding was associated with neither moral disengagement nor social domain knowledge. Also for this behavior other factors may be more relevant. Thornberg and Jungert (2013) found that students who are low in defender self-efficacy are more inclined to act as passive bystanders, even if they display low moral disengagement. Furthermore, students may not intervene in bullying episodes because they have a low sense of safety at school (Gini et al., 2008). Hence, the non-intervention in bullying situations may stem not from moral evaluations of behaviors, but from other elements. Moreover, Obermann (2011) suggested a distinction between unconcerned passive bystanders (high in moral disengagement) and guilty passive bystanders (low in moral disengagement), which may equalize the effect of each other in a sample if this distinction is not addressed in the analysis. Finally, we used measures focused on the moral evaluation of aggressions and bullying. By developing measures able to assess how much *not intervening* in bullying situations is perceived as a moral transgression (due to withdrawing from prosocial behaviors) may detect some associations between the two moral dimensions we investigated and passive bystanding.

### Limitations and Future Directions of Research

The main limitation of this study consists in the cross-sectional nature of the data that does not allow to draw strong conclusions on the direction of the associations. Notwithstanding, to our knowledge this is the first study investigating the possible relation between the organization of moral knowledge in domains and moral disengagement mechanisms, also in connection with three participant behaviors in bullying. In this perspective, our results contribute to the literature on morality clarifying that the two moral dimensions are actually distinct constructs, only moderately associated, and with different relations with youth’s behaviors in bullying. We interpreted this distinction mainly with the *a priori* and *a posteriori* functioning of these constructs, but we need more studies investigating the possible intersections of different dimensions of morality, also in relation to moral emotions.

Lastly, our results can contribute to the literature on anti-bullying interventions, as we confirmed that moral disengagement is a moral cognitive dimension affecting the perpetration of bullying more than others. Therefore, we need more anti-bullying interventions addressing this aspect.

## Data Availability Statement

Due to limitations from the Privacy Law restrictions and the participants’ ethical consents, the raw data cannot be made accessible out of the research team. Aggregated analyses results, however, can be requested from the corresponding author.

## Author Contributions

SC made substantial contributions to the conception of the work, the acquisition, analysis, and interpretation of data for the work, drafting the work, and revising it critically for important intellectual content, provided approval for publication of the content, and agreed to be accountable for all aspects of the work in ensuring that questions related to the accuracy or integrity of any part of the work were appropriately investigated and resolved. JF made substantial contributions to the conception of the work, interpretation of data for the work, drafting the work and revising it critically for important intellectual content, provided approval for publication of the content, and agreed to be accountable for all aspects of the work in ensuring that questions related to the accuracy or integrity of any part of the work were appropriately investigated and resolved. HF made substantial contributions to the conception of the work, drafting the work, and revising it critically for important intellectual content, provided approval for publication of the content, and agreed to be accountable for all aspects of the work in ensuring that questions related to the accuracy or integrity of any part of the work were appropriately investigated and resolved.

## Conflict of Interest

The authors declare that the research was conducted in the absence of any commercial or financial relationships that could be construed as a potential conflict of interest.

## Publisher’s Note

All claims expressed in this article are solely those of the authors and do not necessarily represent those of their affiliated organizations, or those of the publisher, the editors and the reviewers. Any product that may be evaluated in this article, or claim that may be made by its manufacturer, is not guaranteed or endorsed by the publisher.
