# Supplementary material for: Two Dimensions of Moral Cognition as Correlates of Different Forms of Participation in Bullying
Source: Front Psychol. 2022 Feb 18;12:768503. doi: 10.3389/fpsyg.2021.768503 (PMC8896117; doi:10.3389/fpsyg.2021.768503)
Supplement: Supplementary file 1 [file Table_1.docx]

**Table**

*Descriptive statistics (Means and Standard Deviations) of the observed variables - Parcels of Moral Disengagement and Socio-conventionality of moral rules, and scores of the Roles of participation in bullying.*___________________________________________________________________

Parcel n M SD
**Moral disengagement**
Justification 269 2.08 0.82
Euphemistic labeling 271 1.85 0.84
Advantageous comparison 270 2.53 1.15
Responsibility replacement 273 1.88 1.09
Victim blame 269 2.26 0.78
Dehumanization 274 2.00 1.02
Consequence minimization 275 1.59 1.12
**Socio-conventionality of moral rules**Verbal violence Teacher permission 276 1.49 0.60
Verbal violence Principal permission 276 1.58 0.67
Verbal violence Context dependent 276 1.47 0.55
Physical violence Teacher permission 276 1.40 0.55
Physical violence Principal permission 276 1.49 0.67
Physical violence Context dependent 276 1.37 0.50
Relational violence Teacher permission 275 1.37 0.58
Relational violence Principal permission 275 1.50 0.68
Relational violence Context dependent 275 1.53 0.61

Roles in bullying n M SD
Perpetrator 276 -0.16 0.81
Defender of the victim 276 0.19 0.86
Bystander 276 -0.03 0.82

Note. Scale range for the Parcels: Moral disengagement from 1 to 5 (items rated along a 5 point response scale from 1 = strongly disagree to 5 = strongly agree); Socio-conventionality of moral from 1 to 4 (items rated along . a 4 point likert scale from 1 = totally wrong to 4 = totally right). Higher scores indicated higher moral disengagement and higher perceived socio-conventionality.
